# Azilsartan as an antihypertensive treatment in Japanese children under 6 years old: A phase 3 open‐label long‐term study

**DOI:** 10.1111/ped.70284

**Published:** 2025-12-08

**Authors:** Shuichi Ito, Masakazu Miyamoto, Yuki Mizuta, Kenkichi Sugiura, Taisuke Kondo

**Affiliations:** ^1^ Department of Pediatrics Yokohama City University Yokohama Kanagawa Japan; ^2^ Takeda Development Center Japan Takeda Pharmaceutical Company Limited Osaka Japan

**Keywords:** angiotensin receptor antagonist, blood pressure, child, essential hypertension, hypertension

## Abstract

**Background:**

Pediatric hypertension presents unique challenges in its management and treatment. A previous study demonstrated the safety and efficacy of azilsartan in children 6–15 years old, but evaluation in children under 6 years old has not been conducted.

**Methods:**

This phase 3, open‐label study assessed the safety and efficacy of azilsartan in Japanese pediatric patients (2 to <6 years old) with hypertension. Patients received azilsartan once daily for 52 weeks, starting with 0.1 mg/kg and titrating up to a maximum of 0.8 mg/kg if target blood pressure (BP) had not been achieved. The primary objective was the incidence of treatment‐emergent adverse events (TEAEs) and other safety parameters.

**Results:**

All nine patients who received azilsartan experienced TEAEs; most (88.9%) were mild‐to‐moderate in severity. Three patients (33.3%) experienced drug‐related TEAEs (acute kidney injury, anemia, and renal impairment). Acute kidney injury was the only serious drug‐related TEAE, which resolved following treatment interruption. There were no deaths or study discontinuations due to TEAEs. At weeks 12 and 52, mean (SD) changes in systolic BP from baseline (last observation carried forward) were −8.0 (6.7) and −10.9 (8.3) mmHg, respectively. The mean changes in diastolic BP from baseline were −10.9 (11.0) and −14.8 (8.4) mmHg, respectively.

**Conclusion(s):**

Azilsartan was generally well tolerated and associated with sustained BP reductions over 52 weeks in nine Japanese pediatric patients with hypertension. No new safety signals were identified. Our results indicate that azilsartan can be a therapeutic option for managing pediatric hypertension, potentially improving long‐term cardiovascular outcomes.

## INTRODUCTION

Uncontrolled elevated blood pressure (BP) in children can result in severe complications.^1^, ^2^ Elevated BP during childhood increases the risk of adult hypertension and metabolic syndrome, and individuals are also more likely to have persistent hypertension.^3^, ^4^, ^5^, ^6^ A meta‐analysis of 47 publications up to 2018 revealed a 4.0% global pooled prevalence of hypertension in children and adolescents up to 19 years old.^7^ In children between the ages of 6 and 9 years, the prevalence was similar (4.1%), nearly doubling during the period from 2000 to 2015.^7^

There are two types of hypertension. Essential hypertension is characterized by elevated BP without symptoms, often linked to a mild degree of hypertension, obesity, and family history.^2^ It is generally more common in adolescent children and adults than in younger children.^2^ Secondary hypertension is due to a specific underlying condition and is common in younger children.^2^ The most common causes of secondary hypertension in young children are renal and renovascular diseases, which account for 60%–80% of cases.^2^, ^8^

The Japanese Society of Hypertension recommends pharmacological therapy when dietary/lifestyle changes are inadequate in children with essential hypertension.^2^ For children with secondary hypertension, pharmacological therapy is recommended as a first‐line option. Only five antihypertensive medications are approved for children and adolescents in Japan: valsartan, candesartan, enalapril, lisinopril, and amlodipine; among these, only candesartan and enalapril are indicated in children under 6 years of age.^2^ None of these have a formulation for pediatric patients. In the United States, while overall options are still limited, at least two additional antihypertensive drugs (hydrochlorothiazide and valsartan) are approved for use in children under 6 years of age, as well as candesartan and enalapril.^9^, ^10^, ^11^ To prevent complications associated with pediatric hypertension and hypertension that continues into adulthood, it is important to evaluate novel or existing antihypertensive drugs in a pediatric population and manage elevated BP early in childhood. Both the angiotensin II receptor blockers (ARBs) candesartan and valsartan have been shown to effectively lower BP in children under 6 years old.^12^, ^13^

Azilsartan is an ARB that selectively binds with high affinity to the angiotensin II type 1 receptor and is more potent than existing ARBs.^14^ In a phase 3 study, effective reductions in BP were observed in adults with hypertension treated with azilsartan.^15^ Another phase 3, open‐label study in children who were 6–15 years old and treated with azilsartan reported antihypertensive effects and a manageable safety profile at doses up to 20 mg in those who weighed less than 50 kg and up to 40 mg in those who weighed at least 50 kg.^16^, ^17^ Azilsartan had similar safety profiles in both studies.^15^, ^16^, ^17^ In Japan, azilsartan is approved for the treatment of hypertension in adults (10–40 mg once daily [QD]) and in children over 6 years old (2.5–40 mg QD). The primary objective of the current study was to evaluate the long‐term safety of azilsartan in Japanese children with hypertension between the ages of 2 and 6 years.

## MATERIALS AND METHODS

### Study design

**FIGURE 1:**
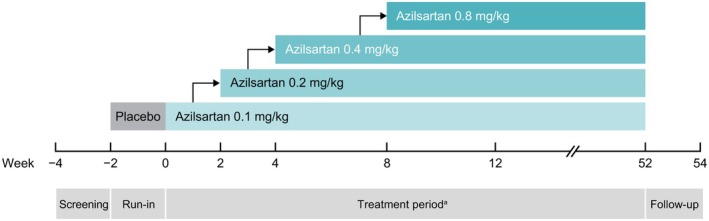
Study design. ^a^During the treatment period, if patients did not achieve target BP and there were no safety or tolerability concerns, azilsartan was titrated every 2 or 4 weeks up to week 8 in a stepwise manner. Dosage could be reduced at the investigator's discretion if there were any safety or tolerability concerns with the titration. Beyond week 8, azilsartan could be titrated in a similar manner if the target BP was not achieved and there were no safety or tolerability concerns. BP, blood pressure.

This was a phase 3, open‐label, multicenter study designed to evaluate the safety and efficacy of azilsartan QD for 52 weeks in young pediatric patients with hypertension. The study consisted of a screening period (weeks −4 to −2), run‐in period (weeks −2 to 0), treatment period (weeks 0 to 52), and follow‐up period (weeks 52 to 54), which gave a total study duration of 56 weeks (Figure 1). The study was conducted at 19 sites in Japan and is registered on ClinicalTrials.gov (NCT04668157). The clinical study protocol including any amendments, patient consent form, and other study‐related documents were reviewed and approved by the institutional review boards (IRBs) at all study sites. The study design, including sample size and endpoints, was agreed upon by the Pharmaceuticals and Medical Device Agency in Japan.

### Patient population

Japanese patients with a diagnosis of essential or secondary hypertension who were between 2 and <6 years old were eligible for inclusion. Essential hypertension was defined as an office sitting systolic BP (SBP) or diastolic BP (DBP) in the 95th percentile or above by age and sex without concomitant hypertensive organ damage (Table S1).^18^ Secondary hypertension was defined as an office sitting SBP or DBP in the 90th percentile or above by age and sex with concomitant chronic kidney disease, diabetes, heart failure, or hypertensive organ damage.

Office sitting BP was measured in the morning before administration of the study drug. If scheduled alongside blood draws, BP was measured beforehand. Patients were instructed to avoid eating or bathing (within 1 h) and caffeine intake (within 30 min) before measurement. After resting in a seated position, BP was measured three times using an appropriately sized cuff (40% of arm circumference) on the same arm, held at heart level. The same device and (where possible) the same investigator or study collaborator were used throughout the study. The arithmetic mean of the three readings was used.

To be included, patients had to be able to swallow the study drug and have a parent or legal guardian who was able to comply with protocol requirements. Patients were also required to have a body weight of not less than two standard deviations (SDs) below the mean population reference by age.^19^ Patients who had undergone kidney transplantation were eligible if the graft was functionally stable (estimated glomerular filtration rate [eGFR] ≥30 mL/min/1.73 m^2^) for at least 6 months before screening.

Key exclusion criteria included previous treatment with azilsartan; poorly controlled hypertension (office sitting SBP ≥22 mmHg above and/or DBP ≥17 mmHg above the 95th percentile of the reference BP values by age and sex); malignant or accelerated hypertension; severe renal dysfunction (eGFR <30 mL/min/1.73 m^2^), renovascular disease affecting one or both kidneys, or current dialysis; treatment compliance <70% or >130% during run‐in; history or signs/symptoms of serious cardiovascular, hepatobiliary, gastrointestinal, endocrine, hematologic, immunologic, urogenital, or psychiatric disease, cancer, or any other disease that may adversely affect the patient's health or confound the study results; previous or concurrent clinically significantly abnormal 12‐lead electrocardiogram (ECG) findings; poorly controlled diabetes mellitus (HbA_1c_ > 9.0%) at screening; use of any investigational drug within 30 days of screening; and current participation in another clinical/post‐marketing study.

### Treatment

Eligible patients received placebo QD as a single‐blinded treatment at the start of the run‐in period (week −2) and were allowed to enter the treatment period if their BP met the inclusion criteria after a minimum of 1 week. The run‐in period was extended to 4 weeks for patients previously treated with any antihypertensive medication if their BP did not meet the inclusion criteria after 2 weeks. Patients taking concomitant antihypertensive medications (excluding renin‐angiotensin system [RAS] inhibitors) before the run‐in period were allowed to continue one of these medications in addition to azilsartan during the treatment period if deemed necessary by the investigators. Patients taking RAS inhibitors were still eligible if they could safely discontinue them by the start of the run‐in period. The use of RAS inhibitors was not allowed during the treatment period.

During the treatment period, patients received open‐label azilsartan based on their body weight at an initial dose of 0.1 mg/kg QD (not exceeding 2.5 mg/day). At week 2, 4, or 8, the dose of azilsartan could be stepwise titrated to 0.2 mg/kg QD (not exceeding 5 mg/day), 0.4 mg/kg QD (not exceeding 10 mg/day), and 0.8 mg/kg QD (not exceeding 20 mg/day) if the patient had not achieved the target BP (<95th percentile for essential hypertension or < 90th percentile for secondary hypertension) and there were no safety or tolerability concerns. An unscheduled titration visit at week 6 was also permitted at the discretion of the investigator. If the target BP was still not achieved beyond week 8 and the patient was not on the maximum dose (0.8 mg/kg), a stepwise titration could be performed following the same approach and conditions mentioned above.

Concomitant antihypertensive medications (excluding RAS inhibitors), steroids and/or central nervous system (CNS) or non‐CNS stimulants (which are known to affect BP) were permitted up to week 12 of the treatment period. However, only one antihypertensive medication was permitted, and no dosage changes were allowed for steroids and/or CNS or non‐CNS stimulants during this period. After week 12, the addition of antihypertensive medications (excluding RAS inhibitors) or change in doses of their existing concomitant antihypertensive medications was permitted if target BP was not achieved with the maximum dose of azilsartan. Azilsartan could be down‐titrated at the discretion of the investigator if there were concerns about safety or tolerability during the treatment period. Dose reduction or interruption, as deemed necessary by the investigators owing to safety or tolerability concerns, was performed on the concomitant antihypertensive medications first before azilsartan.

### Endpoints

The primary objective was to evaluate the safety profile of azilsartan. Endpoints included the incidence of treatment‐emergent adverse events (TEAEs; defined as any adverse event occurring after initiating azilsartan until the end of the follow‐up period), resting 12‐lead ECG parameters, laboratory assessments, and growth (weight, height, BMI) and vital sign (office sitting pulse rate and home sitting BP) measurements. TEAEs were coded using the Medical Dictionary for Regulatory Activities (MedDRA, version 26.0). Adverse events of special interest (AESI) were also assessed (hypotension‐, renal dysfunction‐, and hyperkalemia‐related TEAEs). Hypotension was determined by the investigators without a specific cutoff value.

The secondary objective was to evaluate the efficacy of azilsartan and endpoints included the changes in office sitting BP at weeks 12 and 52 from baseline, and the proportions of patients who achieved the target BP at weeks 12 and 52 (responders).

Assessments of TEAEs, physical examination, concomitant medications, and BP were performed throughout the 56‐week study period.

### Statistical analysis

The study planned to enroll 10 patients to enter the treatment period, based on enrollment feasibility rather than by statistical power calculation. The safety analysis and full analysis sets consisted of all patients who were enrolled and received at least one dose of azilsartan during the treatment period. Continuous variables were summarized using descriptive statistics (number, percentage, mean, and SD). Categorical variables were summarized using the number and percentage of patients. TEAEs were summarized by frequency distribution of System Organ Class and Preferred Term. The last observation carried forward (LOCF) method was used for the changes in BP from baseline and proportions of patients achieving target BP at weeks 12 and 52. A descriptive subgroup analysis was conducted to summarize the proportions of patients at weeks 12 and 52 who achieved target BP according to demographic and clinical characteristics (age, sex, weight, eGFR, steroid use [at the start of the treatment period], and RAS inhibitor use [before the run‐in period]). All analyses were performed using SAS version 9.4 (SAS Institute Inc., Cary, NC, USA).

## RESULTS

### Patient characteristics

**FIGURE 2:**
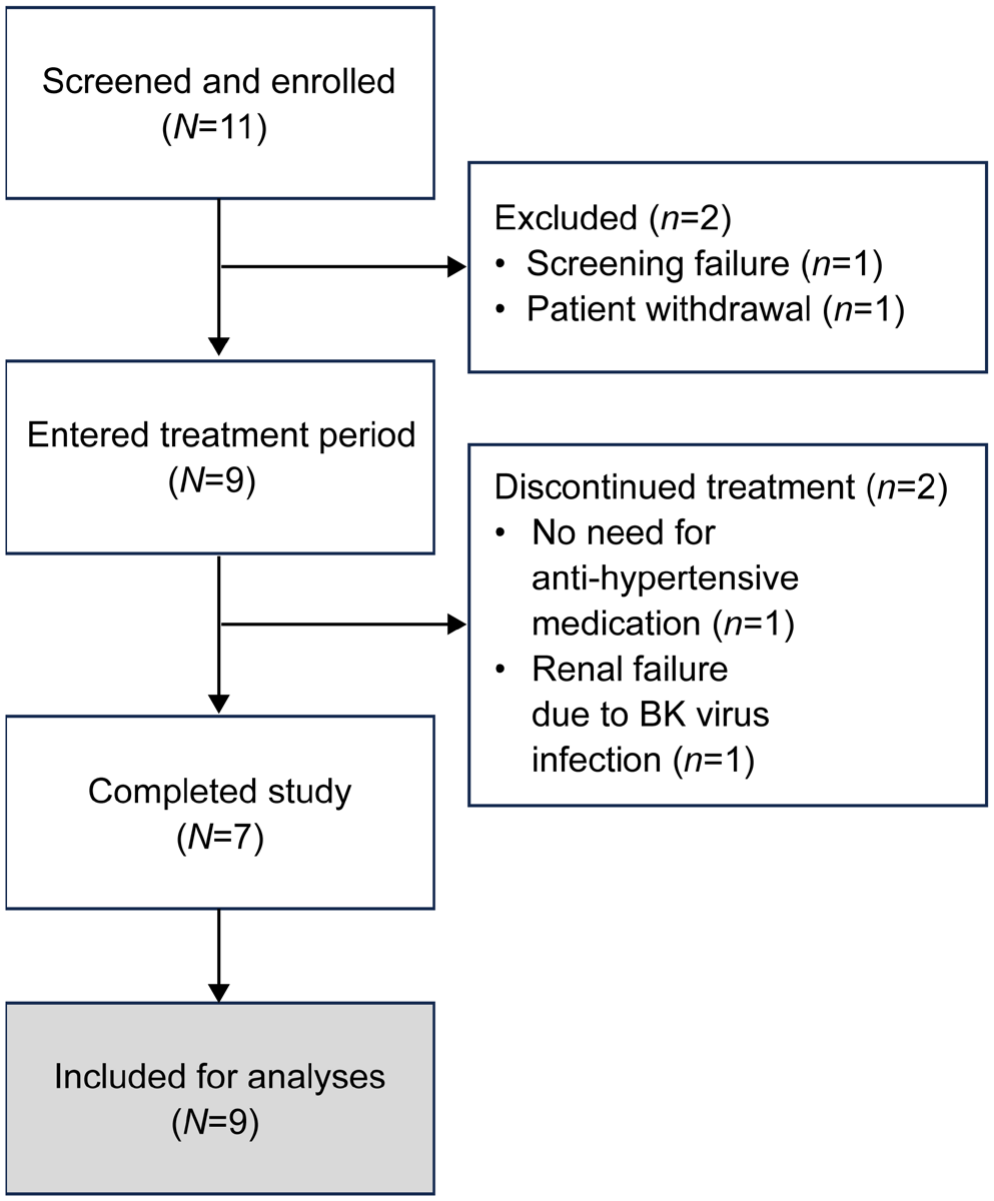
Patient disposition.

Between May 17, 2021, and December 28, 2023, 11 patients were enrolled. In total, nine patients (81.8%) entered the treatment period (one was excluded for not meeting all the inclusion criteria and one withdrew from the study). Seven patients (77.8%) completed the treatment and follow‐up periods. One patient discontinued azilsartan treatment after 200 days because antihypertensive medication was no longer required and another discontinued after 110 days due to renal failure caused by a BK virus infection (Figure 2). However, both patients were included for subsequent analyses. Two other patients had at least one significant protocol deviation (unable to visit the study site at week 52 owing to COVID‐19 and registration failure owing to a technological issue) but were included in the safety and efficacy analyses.

Mean (SD) age at informed consent was 3.4 (1.1) years and the proportions of female and male sex were similar (Table 1). All patients were diagnosed with secondary hypertension, of whom two (22.2%) had drug‐induced hypertension. Of the remaining seven patients, five (55.6%) had underlying renal and urinary disorders and two (22.2%) had congenital, familial, and genetic disorders (renal aplasia and renal hypoplasia). The patients with drug‐induced hypertension also had underlying renal and urinary disorders. No patient had a history of kidney transplantation, and two received RAS inhibitors before the run‐in period.

**TABLE 1:** Baseline demographics and clinical characteristics.

Characteristic	Overall (*N* = 9)
Age (years), mean (SD)	3.4 (1.1)
Age, *n* (%)	
2	2 (22.2)
3	3 (33.3)
4	2 (22.2)
5	2 (22.2)
Male sex, *n* (%)	5 (55.6)
Weight (kg), mean (SD)	16.0 (4.0)
BMI (kg/m^2^), mean (SD)	16.0 (1.3)
Disease duration (years), mean (SD)	0.6 (0.7)
Type of hypertension, *n* (%)	
Essential hypertension	0
Secondary hypertension	9 (100)
Antihypertensive medications, *n* (%)	
Before the run‐in period	2 (22.2)^a^
At the start of the treatment period	1 (11.1)
Office sitting BP (mmHg), mean (SD)	
SBP	111.0 (7.6)
DBP	67.7 (5.5)
eGFR (mL/min/1.73 m^2^), mean (SD)	99.0 (31.9)

Abbreviations: ARB, angiotensin receptor blocker; BMI, body mass index; BP, blood pressure; CCB, calcium channel blocker; DBP, diastolic blood pressure; eGFR, estimated glomerular filtration rate; RAS, renin‐angiotensin system; SBP, systolic blood pressure; SD, standard deviation.

^a^ Two patients were on RAS inhibitors. One patient received an ARB and another received an ARB and a CCB.

All patients had ≥80% treatment compliance, with a mean (SD) compliance rate of 97.1% (6.4). The mean (SD) duration of azilsartan exposure was 313.3 (92.7) days. At the end of the treatment period, patients were on azilsartan 0.1 mg/kg (*n* = 3), 0.2 mg/kg (*n* = 1), 0.4 mg/kg (*n* = 3), and 0.8 mg/kg (*n* = 2).

### Safety and tolerability profile

All patients experienced at least one TEAE, most of which were mild (*n* = 6 [66.7%]) or moderate (*n* = 2 [22.2%]) in severity (Table 2). One patient had a severe TEAE (acute kidney injury). The most frequently reported TEAE was nasopharyngitis (*n* = 7 [77.8%]), followed by bronchitis, COVID‐19, and gastroenteritis (*n* = 2 [22.2%] each). Three patients (33.3%) experienced drug‐related TEAEs, including acute kidney injury, anemia, and renal impairment (*n* = 1 [11.1%] each) (Table 3). Both anemia and renal impairment were mild in severity. The patient who experienced anemia recovered without changes to treatment administration. In total, six serious TEAEs were reported in four patients and were COVID‐19 (2 [22.2%] events) and acute kidney injury, colitis, coronavirus infection, and proteinuria (1 [11.1%] event each); all resolved within 30 days of onset. Of these serious TEAEs, only acute kidney injury was severe and related to azilsartan as deemed by the investigator. The patient who experienced severe acute kidney injury presented with dehydration due to vomiting and diarrhea. This patient was later diagnosed with gastroenteritis and admitted to hospital owing to increased serum creatinine. The acute kidney injury resolved soon after the gastroenteritis was treated, and the study treatment was stopped; subsequently, azilsartan was not resumed as the patient's BP was found to be under control at the hospital.

**TABLE 2:** Overview of TEAEs (safety analysis set).

	Overall (*N* = 9)	
	*n* (%)	Number of events
Any TEAE	9 (100)	62
Related to study drug	3 (33.3)	4
Severity of TEAE		
Mild	6 (66.7)	59
Moderate	2 (22.2)	2
Severe	1 (11.1)	1
TEAEs leading to treatment discontinuation	0	0
Serious TEAEs	4 (44.4)	6
Related to study drug	1 (11.1)	1
Serious TEAEs leading to treatment discontinuation	0	0
TEAEs resulting in death	0	0
AESI		
Hypotension‐related	1 (11.1)	1
Renal dysfunction‐related	2 (22.2)	4
Hyperkalemia‐related	0	0

Abbreviations: AESI, adverse event of special interest; TEAE, treatment‐emergent adverse event.

**TABLE 3:** Any and drug‐related TEAEs by MedDRA SOC and PT (safety analysis set).

	Any TEAE^a^ (*N* = 9)	Drug‐related TEAE (*N* = 9)
	*n* (%)	*n* (%)
Experienced TEAE	9 (100)	3 (33.3)
Infections and infestations	8 (88.9)	–
Nasopharyngitis	7 (77.8)	–
Bronchitis	2 (22.2)	–
COVID‐19	2 (22.2)	–
Gastroenteritis	2 (22.2)	–
Gastrointestinal disorders	3 (33.3)	–
Abdominal pain	1 (11.1)	–
Colitis	1 (11.1)	–
Constipation	1 (11.1)	–
Diarrhea	1 (11.1)	–
Stomatitis	1 (11.1)	–
Renal and urinary disorders	3 (33.3)	2 (22.2)
Acute kidney injury	1 (11.1)	1 (11.1)
Pollakiuria	1 (11.1)	–
Proteinuria	1 (11.1)	–
Renal impairment	1 (11.1)	1 (11.1)
Skin and subcutaneous tissue disorders	3 (33.3)	–
Dry skin	1 (11.1)	–
Hangnail	1 (11.1)	–
Purpura	1 (11.1)	–
Blood and lymphatic system disorders	1 (11.1)	1 (11.1)
Anemia	1 (11.1)	1 (11.1)

Abbreviations: COVID‐19, coronavirus disease 2019; MedDRA, Medical Dictionary for Regulatory Activities; PT, Preferred Term; SOC, System Organ Class; TEAE, treatment‐emergent adverse event.

^a^ Reported in at least three patients by SOC.

Regarding AESI, one patient experienced mild hypotension and recovered on the same day following treatment interruption. Two patients experienced a total of four renal dysfunction‐related TEAEs (one patient experienced two events of renal impairment and another had acute kidney injury and proteinuria). Of these, acute kidney injury and renal impairment were considered to be causally related to azilsartan by the investigator. The patient who experienced two occurrences of renal impairment recovered after treatment interruption for the first occurrence and without changes to treatment administration for the second occurrence. There were no hyperkalemia‐related TEAEs, no deaths, or TEAEs that led to treatment discontinuation.

No remarkable findings or clinical concerns were observed throughout the 52‐week treatment period regarding laboratory assessments, office sitting pulse rate, home sitting BP, physical examinations, resting 12‐lead ECG, or growth measurements.

### Changes in BP

**FIGURE 3:**
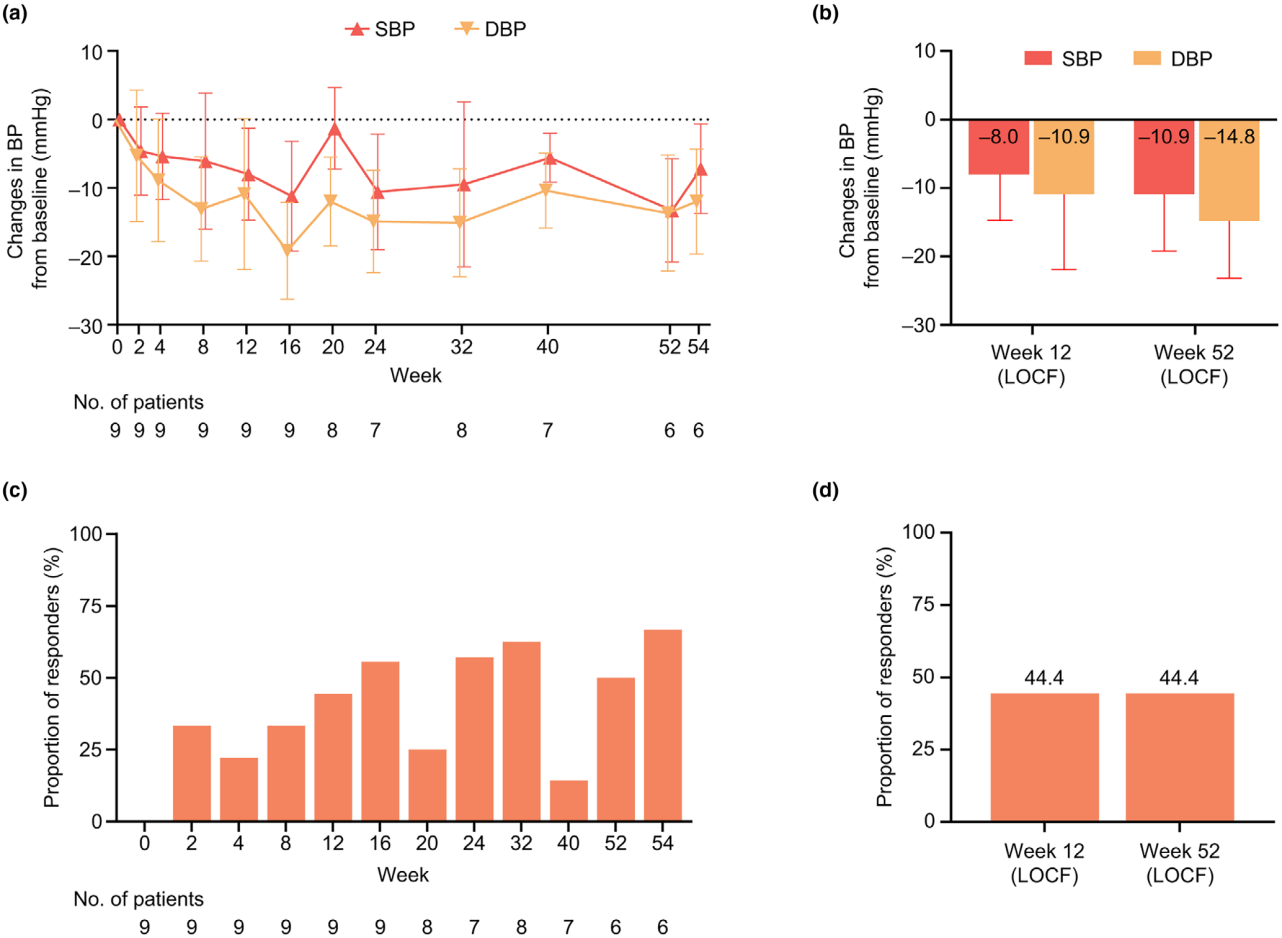
Changes in SBP and DBP, and the proportions of responders^a^. Mean (SD) changes in SBP and DBP from baseline throughout the study period (a) and at weeks 12 and 52 with LOCF for both time points (b). The proportions of patients who achieved target BP (based on age and sex) throughout the study (c) and at weeks 12 and 52 with LOCF for both time points (d). ^a^Patients who achieved target SBP and DBP. BP, blood pressure; DBP, diastolic blood pressure; LOCF, last observation carried forward; SBP, systolic blood pressure; SD, standard deviation.

Mean baseline SBP and DBP were 111.0 mmHg and 67.7 mmHg, respectively, in the full analysis set. In general, BP reductions occurred throughout the treatment period (Figure 3, Figure S1). The mean changes from baseline in SBP and DBP at week 12 (LOCF) were −8.0 mmHg and −10.9 mmHg, respectively. At week 52 (LOCF), the mean changes from baseline were −10.9 mmHg and −14.8 mmHg, respectively.

Similar to the office sitting BP, reductions in mean values from baseline were observed at week 12 in home sitting SBP (−8.5 mmHg) and DBP (−10.0 mmHg) blood pressure.

At week 2, three out of nine patients achieved target BP (responders) (Figure 3). At both weeks 12 and 52 (LOCF), four out of nine patients achieved target BP (44.4%). A descriptive subgroup analysis of proportions of patients achieving target BP according to their demographic and clinical characteristics is available in Table S2.

## DISCUSSION

This phase 3, open‐label study evaluated the clinical profile of azilsartan over 52 weeks in young pediatric patients (2 to <6 years old) with hypertension. This is the first study to investigate azilsartan in this population, for which limited pharmacological safety and efficacy data are available. Azilsartan was well tolerated with no treatment discontinuations due to TEAEs, and no new safety signals were detected. The safety profile was consistent with previous azilsartan studies conducted in adults^15^ and older children (6–15 years old).^16^, ^17^ Mean reductions in BP occurred as early as 2 weeks following treatment and were maintained for up to 1 year. Few drugs are approved globally for treating hypertension in this young age group;^8^ based on our trial data, azilsartan received regulatory approval for this patient population in Japan in August 2024.

The safety profile of azilsartan was the primary endpoint. Most TEAEs were mild or moderate in severity with the most frequent TEAE being nasopharyngitis (*n* = 7 [77.8%]). All drug‐related TEAEs (acute kidney injury, anemia, and renal impairment) were also observed in older children treated with azilsartan.^16^, ^17^ Laboratory tests, physical examinations, ECGs, and growth measurements revealed no clinical concerns or remarkable findings.

In our study, hypotension was reported in one patient and a renal dysfunction‐related AESI was reported in two patients. These were also observed in the azilsartan study in older children.^16^, ^17^ In patients whose renal function may depend on the RAS (e.g., patients with severe congestive heart failure, renal artery stenosis, or volume depletion), inhibition of RAS by ARBs has been associated with renal impairment, including rare cases of acute kidney injury.^11^, ^20^, ^21^ The patient who experienced severe acute kidney injury in our study had other risk factors (dehydration from vomiting and diarrhea due to gastroenteritis), which may have exacerbated the kidney injury. Nonetheless, this AE resolved soon after stopping treatment. These findings underscore the importance of monitoring for potential renal complications in young patients during ARB treatment. Few clinical trials have evaluated antihypertensive agents in young children (<6 years old) with hypertension^12^, ^13^, ^22^ and none have been conducted in Japanese pediatric patients. Our data thus provide valuable insights for physicians and patients.

In our study, patients with renal artery stenosis in both kidneys (bilateral), or patients with unilateral renal artery stenosis who only had one kidney were excluded owing to the known risk of renal function deterioration when treating renal artery stenosis (particularly bilateral) with RAS inhibitors.^23^ Indeed, while azilsartan may be considered for patients with severe hypertension, its use in renovascular hypertension should generally be avoided, consistent with other RAS inhibitors. Patients with uncontrolled hypertension were also excluded from our study, given that such patients often require urgent intervention that is not conducive to having a placebo run‐in period.

The efficacy profile of azilsartan was the secondary endpoint. Clinically meaningful changes in BP were observed from baseline to week 12, a period which had restrictions on concomitant medications, as well as at week 52. In older children with hypertension treated with azilsartan, the mean changes in SBP and DBP from baseline at week 12 were −12.4 and −13.9 mmHg, respectively,^16^, ^17^ which were greater than the BP changes observed in younger children in our current study. A possible explanation for this could be the higher mean baseline SBP (125.7 mmHg) and DBP (72.0 mmHg) in older children compared with our younger population, which would have required a greater reduction in BP to achieve target levels.

Our data are comparable to those of other ARBs reported in similar pediatric patient populations. Similar changes in BP were observed in a 56‐week trial that evaluated candesartan cilexetil in children aged 1 to under 6 years old (*n* = 93),^13^ and in a 26‐week trial that evaluated valsartan in children aged 0.5–5 years old (*n* = 75).^22^ However, nearly twice the proportion of responders (*n* = 88; 77.3%) was seen in another long‐term study that evaluated valsartan in children aged 1–5 years old, compared with our study.^12^ This may have been attributed to differences in study design (i.e., a randomized, double‐blind, 2‐week fixed‐dose treatment period with low‐, medium‐, or high‐dose valsartan; a rerandomized, 2‐week treatment period with initial valsartan dosage or placebo; and a 52‐week open‐label period with valsartan ± hydrochlorothiazide). Additionally, patient eligibility differed (i.e., valsartan was used as add‐on therapy in patients with inadequately controlled hypertension on current treatment). When interpreting the results of our study, it is important to exercise caution when directly comparing findings from other studies, as differences in study design can significantly influence outcomes.

The main limitations of our study include the open‐label and single‐arm design. No statistical inferences were carried out owing to small sample sizes. Further clinical trials with larger cohorts and extended follow‐up are essential to validate our findings. In addition, all patients had secondary hypertension and most had underlying renal or urinary disorders, which may limit generalizability to patients with essential hypertension. However, this is consistent with previous studies demonstrating that most secondary causes of hypertension in young children are renal in origin. Additionally, the study's optional titration design and small sample size prevented the evaluation of the pharmacokinetics and pharmacodynamics of azilsartan, which are important for understanding drug exposure and response in these pediatric populations. Moreover, the statistical power of our study was limited by the small sample size and results should therefore be interpreted with caution.

## CONCLUSIONS

In this small, open‐label study of nine Japanese pediatric patients (2 to <6 years old) with hypertension, azilsartan (up to a maximum dose of 0.8 mg/kg) was generally well tolerated with mostly mild‐to‐moderate TEAEs. No new safety signals were observed over a 52‐week period. Reductions in office sitting SBP and DBP were also maintained during the study. Overall, our findings suggest that azilsartan can be a new pharmacological treatment option for young children with hypertension, but larger studies with longer follow‐up are warranted.

## AUTHOR CONTRIBUTIONS

SI, MM, YM, and TK contributed to study conceptualization. KS contributed to data curation, formal analysis, investigation, validation, and visualization. MM, YM, and TK contributed to the investigation. SI, MM, YM, and TK contributed to methodology and project administration. SI contributed to supervision. MM contributed to writing – original draft. All authors contributed to writing – review and editing. All authors have read and approved the final version of the manuscript.

## FUNDING INFORMATION

This study was funded by Takeda Pharmaceutical Co. Ltd.

## CONFLICT OF INTEREST STATEMENT

Shuichi Ito received honoraria from Alexion Pharmaceuticals, AstraZeneca, Bayer Chugai Pharmaceutical, Sanofi Genzyme, and Takeda Pharmaceutical Co. Ltd.; research funding from Chugai Pharmaceutical, Japan Blood Product Organization, and Zenoaq Kogoyo; and consulting fees from Fujiyakuhin, Takeda Pharmaceutical Co. Ltd., and Teijin Pharma. Masakazu Miyamoto is an employee and stockholder of Takeda Pharmaceutical Co. Ltd. Yuki Mizuta, Kenkichi Sugiura, and Taisuke Kondo are employees of Takeda Pharmaceutical Co. Ltd. and hold no stocks.

## ETHICS STATEMENT

All study procedures performed were in accordance with ethical standards of the institutional research committee at each study site (IRB approval numbers: 2021–2, NW2020109 [covering 8 institutions], 1‐03001A, 32–08, 20,010, 2,021,008, 211,004, 2021–31, N2020‐02, 210,013‐B, 22–03, S2021023), the Declaration of Helsinki, Good Clinical Practice (GCP) guidelines and the International Conference on Harmonisation Harmonised Tripartite Guideline for GCP and all applicable regulations. Each patient's parent or legal guardian provided written informed consent before starting any study procedure.

## Supporting information


Table S1.

## Data Availability

The data sets, including the redacted study protocol, redacted statistical analysis plan, and individual participants' data supporting the results reported in this article, will be made available within three months from initial request to researchers who provide a methodologically sound proposal. The data will be provided after its de‐identification, in compliance with applicable privacy laws, data protection, and requirements for consent and anonymization.
