# Supplementary material for: Azilsartan as an antihypertensive treatment in Japanese children under 6 years old: A phase 3 open‐label long‐term study
Source: Pediatr Int. 2025 Dec 8;67(1):e70284. doi: 10.1111/ped.70284 (PMC12709567; doi:10.1111/ped.70284)

Azilsartan as an antihypertensive treatment in Japanese children under 6 years old: A phase 3 open-label long-term study

Shuichi Ito · Masakazu Miyamoto · Yuki Mizuta · Kenkichi Sugiura · Taisuke Kondo

Supporting information

Table S1 Reference SBP/DBP values based on age and sex for essential hypertension (95th percentile) and secondary hypertension (90th percentile).

Age (years)	Male sex		Female sex	
	95th percentile	90th percentile	95th percentile	90th percentile
2	106/59	102/56	106/64	103/60
3	107/62	103/59	108/66	104/62
4	108/66	105/62	109/69	106/65
5	109/69	106/65	110/71	107/67
6	111/71	107/68	111/72	108/69

DBP, diastolic blood pressure; **SBP**, systolic blood pressure.

Reference: The Japanese Circulation Society. JCS 2018 guideline on the clinical examinations for decision making of diagnosis and drug therapy in pediatric patients with congenital heart disease and cardiovascular disorder. 2018. https://www.j-circ.or.jp/cms/wp-content/uploads/2020/02/JCS2018_Yasukochi.pdf. Accessed February 2025.

Table S2 Proportion of patients achieving target blood pressure at Week 12, and Week 52 by demographics and clinical characteristics.

**Characteristic**	**Patients who achieved target BP at Week 12, n/N (%)**	**Patients who achieved target BP at Week 52, n/N (%)**
Age at consent (years)		
≤3	1/5 (20.0)	2/5 (40.0)
≥4	3/4 (75.0)	2/4 (50.0)
Sex		
Male	2/5 (40.0)	2/5 (40.0)
Female	2/4 (50.0)	2/4 (50.0)
Weight at Week 0 (kg)		
<15	2/3 (66.7)	3/3 (100)
≥15	2/6 (33.3)	1/6 (16.7)
Steroid use at the start of the treatment period		
Yes	2/4 (50.0)	2/4 (50.0)
No	2/5 (40.0)	2/5 (40.0)
RAS inhibitor use before the run-in period		
Yes	1/2 (50.0)	1/2 (50.0)
No	3/7 (42.9)	3/7 (42.9)
eGFR (mL/min/1.73 m^2)		
<60	1/1 (100)	1/1 (100)
60–<90	1/2 (50.0)	1/2 (50.0)
≥90	2/6 (33.3)	2/6 (33.3)

Note: n = number of patients who achieved target BP; N = total number of patients with a characteristic.

BP, blood pressure; **eGFR**; estimated glomerular filtration rate; **RAS**; renin–angiotensin system.

Figure S1 Changes in SBP and DBP in individual patients throughout the study period. **DBP**, diastolic blood pressure; **SBP**, systolic blood pressure.

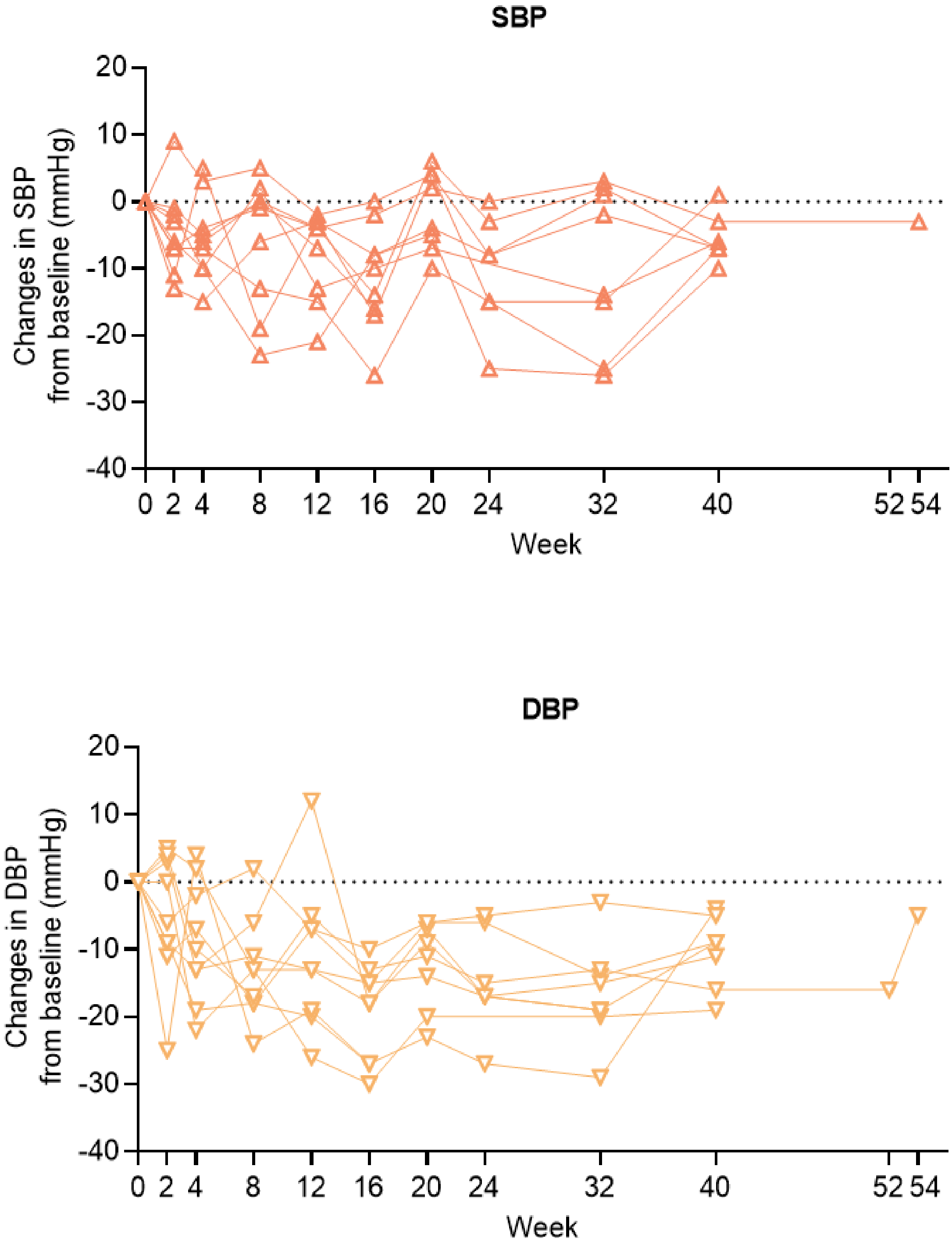

Supplement: Supplementary file 1 — Table S1. [file PED-67-e70284-s001.pdf]
